# Microfluidic model systems used to emulate processes occurring during soft particle filtration

**DOI:** 10.1038/s41598-019-39820-z

**Published:** 2019-02-28

**Authors:** Izabella Bouhid de Aguiar, Martine Meireles, Antoine Bouchoux, Karin Schroën

**Affiliations:** 1 Laboratory of Food Process Engineering, Wageningen University & Research, Wageningen, The Netherlands; 2 Laboratoire de Génie Chimique, Université de Toulouse, CNRS, INPT, UPS, Toulouse, France; 3 Laboratoire d’Ingénierie des Systèmes Biologiques et des Procédés, CNRS, INRA, INSAT, Université de Toulouse, Toulouse, France

## Abstract

Cake layer formation in membrane processes is an inevitable phenomenon. For hard particles, especially cake porosity and thickness determine the membrane flux, but when the particles forming the cake are soft, the variables one has to take into account in the prediction of cake behavior increase considerably. In this work we investigate the behavior of soft polyacrylamide microgels in microfluidic model membranes through optical microscopy for *in situ* observation both under regular flow and under enhanced gravity conditions. Particles larger than the pore are able to pass through deformation and deswelling. We find that membrane clogging time and cake formation is not dependent on the applied pressure but rather on particle and membrane pore properties. Furthermore, we found that particle deposits subjected to low pressures and low g forces deform in a totally reversible fashion. Particle deposits subjected to higher pressures only deform reversibly if they can re-swell due to capillary forces, otherwise irreversible compression is observed. For membrane processes this implies that when using deformable particles, the pore size is not a good indicator for membrane performance, and cake formation can have much more severe consequences compared to hard particles due to the sometimes-irreversible nature of soft particle compression.

## Introduction

Soft particles are in general termed deformable when assuming different shapes upon a stimulus, and compressible when expelling solvent in response to an external force^1,2^. Although these effects are studied, they are often disregarded in technical process designs that revolve around the ‘particle size’. A process in which disregarding deformation and compression can have major effects is membrane separation, since particles larger than the pore size may end up in the permeate.

Membrane filtration is a process in which many types of soft particles can be present such as proteins^3,4^, cells^5^ and sludge flocs^6,7^. Although membrane filtration is used for various separations, ranging from microfiltration to reversed osmosis, and even gas separation, in most of these processes, particle properties play an important role. This can be either in relation to the pore size that particles may or may not be able to pass, or as part of an accumulation layer that forms on top of the membrane leading to increased resistance against mass transfer, and influences on solute transmission.

It is often assumed that particles smaller than the pore size are able to pass while particles that are larger are retained^8^. Please note that this reasoning holds for membranes with uniform pores; in reality most membranes have a pore size distribution, which makes the situation even more complex^9^. Furthermore, the previously described gate-keeper effect of pores holds for hard particles, whereas many particles are compressible and deformable, even to such an extent that depending on the applied driving force, they can be pushed through pores that are smaller than the original particle size^10^. This phenomenon has also been observed in emulsion filtration in which deformable liquid droplets were able to pass membrane pores that were considerably smaller^11,12^.

This has various implications for membrane process design. If the soft particle is to be concentrated, it is desired that the permeate is free of soft particles, but with deformable particles this is not guaranteed based on their original size^10^. If the purity of the permeate is not a problem and the presence of soft particles can be tolerated, even then their presence can be a problem if e.g. backwashing is used. During backwashing the flux direction is reversed from permeate to feed side to remove particle deposits from the top of the membrane^13,14^, but this can also lead to particles being strongly pushed into the membrane, and consequently clogging it. It is clear that the link between membrane pore size or molecular weight cutoff of the filtered component is not that straight-forward to make, and in practice a lot of trial and error is involved^15,16^. The reason for this could be deformability of particles, and that is why we have made this the focal point of this paper, investigating effects inside the membrane as well as on top of it in the so-called cake layer.

Although studies have been done to obtain information on cake formation in filtration, the great majority assess cake formation (so particle accumulation on top of the membrane) in an indirect manner, by measuring changes in flux, and sometimes conductivity^17,18^. These studies generate important information for the overall process but lead not necessarily to mechanistic understanding of local behavior. Further, a number of *in situ* observation techniques have been developed mostly focusing on surface visualizations. We specifically mention Fane *et al*. who developed Optical Coherence Tomography Imaging for visualization of fouling layers as function of time, which has led to new insights in regard to deposition of oil droplets^19,20^. When considering processes occurring inside a membrane, to the best of our knowledge, no techniques are available.

In the last years, microfluidic devices have been used to observe the behavior of particles in systems simulating membrane filtration^21–23^. In previous work we used them to elucidate the behavior of hard particles^24^; soft particle deposition and transmission is still an important and missing element. In the current paper, we use microfluidic methods to observe soft particle deposition and cake behavior for particles that are ‘larger’ than the pore size. The microfluidic devices are composed of an array of parallel channels and we use pAAm microgels as soft particles. We use constant pressure until a cake is observed on top of the channels. Clogging time, clog existence time and cake volume as function of pressure are measured. We also use a microfluidic device mounted on a high-speed centrifuge to emulate the pressure effects. We find that the clogging time is rather independent of applied pressure due to a combined effect of increased number of particles at high flux, and the higher pressure facilitating particle deformation/compression. The centrifuge deposit volume reduction as function of pressure is reversible as long as the particles are not too deformed due to high applied g-forces.

## Material and Methods

### Microgel synthesis and characterization

For the microfluidic filtration experiments, we use tailor-made micrometer-sized polyacrylamide (pAAm) microgels synthesized by us. The microgels are produced by emulsion templating as described in detail in previous work^25^. The particle sizes range from 3 µm to 31 µm with a Sauter mean diameter (D^2,3^) of 10 µm. Commercially available Sephadex G100 microgels were used in some of the centrifugation experiments; sizes range from 35 µm to 100 µm with a D^2,3^ of 64 µm. Size distributions were measured by laser diffraction (Malvern Mastersizer 3000).

### Microfluidic devices

**Figure 1:**
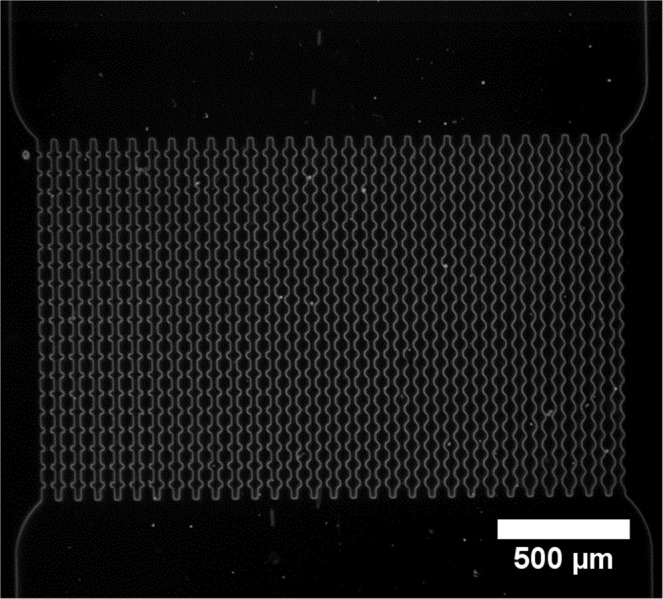
Optical microscopy image of the array of channels that compose the microfluid device at 2.5x magnification. There are 30 parallel channels with five different entrance angles. From left to right: 0°, 20°, 35°, 45°, 55° (see Fig. 2a).

**Figure 2:**
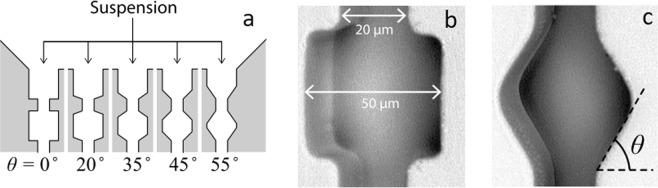
(**a**) Schematic representation of the channels and their different entrance angles, (**b**) internal dimensions of the constrictions, channel depth is 40 µm, (**c**) constriction representing the location where the angles are measured. Reproduced with permission from Nature (van de Laar *et al*.^26^).

The microfluidic device used for the filtration experiments was made by soft lithography. The device is composed of 30 parallel channels and each channel has 19 constrictions along its length (Fig. 1). The channels have 5 different entrance angles (6 channels for each angle) varying from 0° to 55°^26^; Fig. 2 shows the different angles and the dimensions of the channel constrictions.

The channels are positioned in a dead-end configuration and the device has an inlet and an outlet. The devices are connected via Teflon tubing to a pressure controller (Elveflow OB1-MK3) and placed under an optical microscope. Bright field and phase contrast configurations were used. For the filtration experiments we flow a suspension of microgels of approximately 0.1%vol in water at three different pressures; 50, 100 and 150 mbar with precision of 0.1 mbar. Flow of microgel suspension continues until all the channels are clogged; images are taken at a frame rate of 4 fps. After all channels are clogged we keep the flow of microgel suspension to form a cake, or in other words, microgels accumulate on top of the channels. When a cake is formed, we vary the applied pressure to observe compression and relaxation of the cake.

### Centrifugation

**Figure 3:**
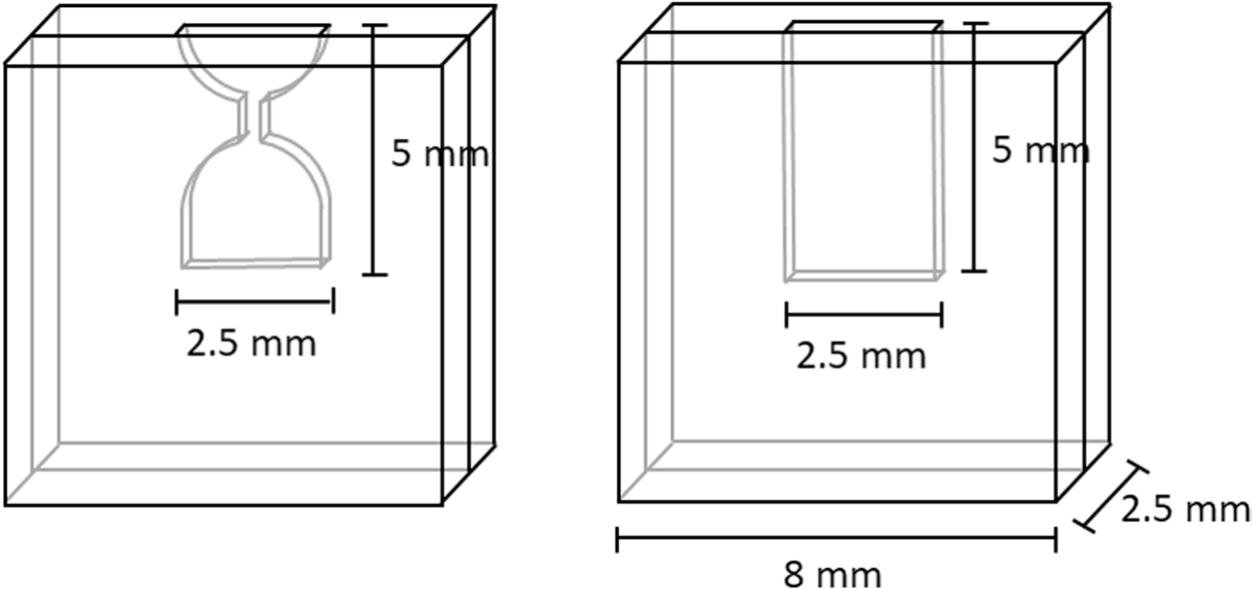
Microcentrifuge microchip designs. Thickness of the chambers is 100 µm.

We also centrifuged the microgel suspension while in a microfluidic device (see Fig. 3) by using a microcentrifuge coupled with an optical microscope, as described in previous work by Krebs *et al*.^27^. Images are taken with a highspeed camera once per rotation and the images are analyzed to determine the (compression/deformation) behavior of the microgels during centrifugation. The centrifugation speed applied varied from 12 g to 1058 g. After rotation stops, images are taken to assess relaxation of the deposited microgels. The following two chamber designs were used (Fig. 3).

For easier comparison between the centrifugation and the microfluidic filtration results, we calculated the approximate pressure at the bottom of the centrifugation chamber for each centrifugation speed used. We first calculated the approximate mass of the microgels from their density and chamber volume (considering a volume fraction of 1) and used this value to calculate the force. We then calculated the pressure by dividing the force by the area of the bottom of the chamber as can be seen in Eq. 1.

1 $$P=\frac{V\rho g{n}_{g}}{A}$$

where *P* is the pressure at the bottom of the microchip chamber, *V* is the volume of the microgel deposit, *ρ* is the density of the microgels, *g* is gravitational constant, *n*_*g*_ is the number of g’s in a determined situation and *A* is the area of the bottom of the microchip chamber.

### Image analysis

The images from microfluidics experiments are treated with home-made matlab scripts, that are available upon request. For the clogging experiments, we manually select the area of the constriction where the clog happens in each channel. The code then tells us at which frame of the stack of images, the selected area is filled with microgels. The code uses black and white pixel differentiation to perform the task. For the centrifuge experiments, we manually select the area of deposits and analyze the images with the software ImageJ^28^.

Throughout the paper, we will analyze the images of the microgel deposits considering only the 2D projection of them. For this reason we will refer in the next sections to cake and deposit areas and not volume. For the cake relaxation experiments, the cake area is measured manually with the help of ImageJ software^28^.

## Results and Discussion

### System clogging time

**Figure 4:**
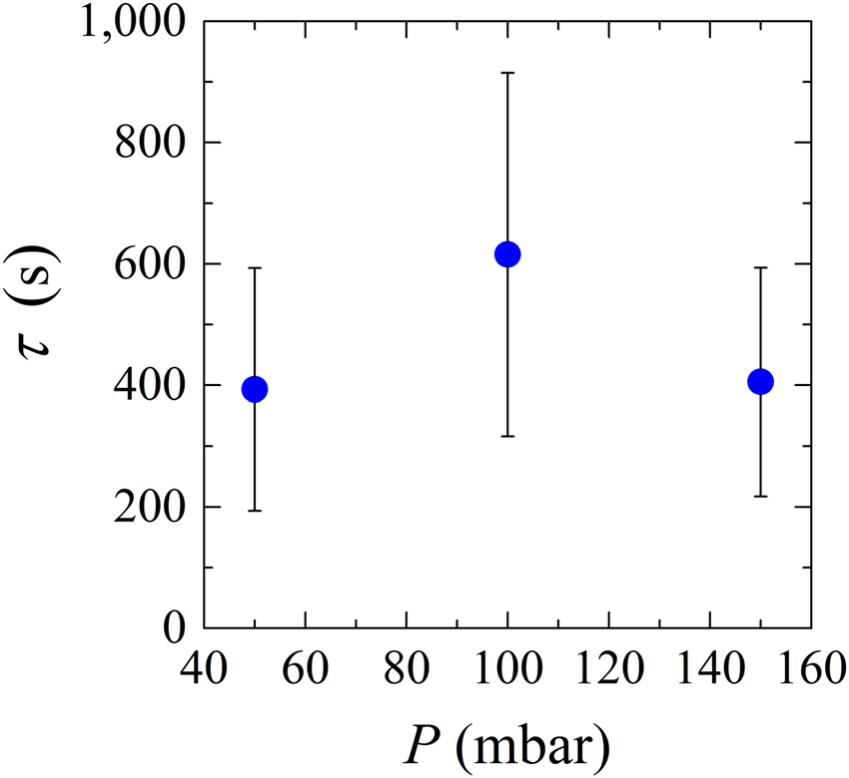
System clog time (*τ*) as a function of pressure (*P*).

Microfluidic devices are used to study the behavior of microgels suspension in filtration systems. In our case, the microgels in suspension are larger than the pore constrictions but are able to modify their conformation to go through the pores. When the microgel suspension flows through the microfluidic device, the pores get blocked one by one by individual particles until the whole system is blocked. We consider the system blocked when there is at least one microgel stuck in every single microchannel. The complete system clogging time was measured as function of the applied pressure. Please note that the device clogging time was taken as the moment at which all channels contained at least one trapped microgel (Fig. 4), although this does not mean that the channels were permanently clogged (see section clog stability).

If the clogging time would be a function of the number of particles of given size and deformability passing a constriction, we would expect a decrease in clogging time with applied pressure (increasing overall flux leads to higher supply of particles). When observing the results presented in Fig. 4, this is clearly not the case. At 50 mbar, the majority of the channels clog at the first constriction (results not shown), so the particles are not pushed through the pore constrictions and clog the channels as soon as they reach them. At 100 mbar, more particles are pushed into the channel and go all the way through the channels, either not blocking them, or blocking deeper in the pore (results not shown). Due to a higher flux at 150 mbar, it is more likely that bigger particles will arrive at the entrance of the channels due to the higher flux, and the propensity to clog increases because these particles are less likely to be pushed all the way through the pores due to their larger size. The large error bars are not a result of unstable clogs but a result of the particle size distribution in combination with process conditions that dictate the deformation behavior, and is linked to clogging probability.

As the channels in the microfluidic devices have a variety of entrance angles, we verified if the channels entrance angles had an impact on the channel’s clogging time and found that this is not the case as expected for hard particles smaller than the constriction^25^. An influence would be expected when particles are closer to the channel/pore size and the angles may facilitate deformation, which is apparently not the case, or when clusters of small particles are responsible for clogging^26^. To be complete, when using these devices in earlier work, we found a very week dependency of clogs being formed at a neighboring pore^24^; therefore we expect this not to have played a significant role in the current work.

To understand the behavior of microgels in filtration systems in greater detail, we focused on clog existence time and cake behavior.

### Clog stability

**Figure 5:**
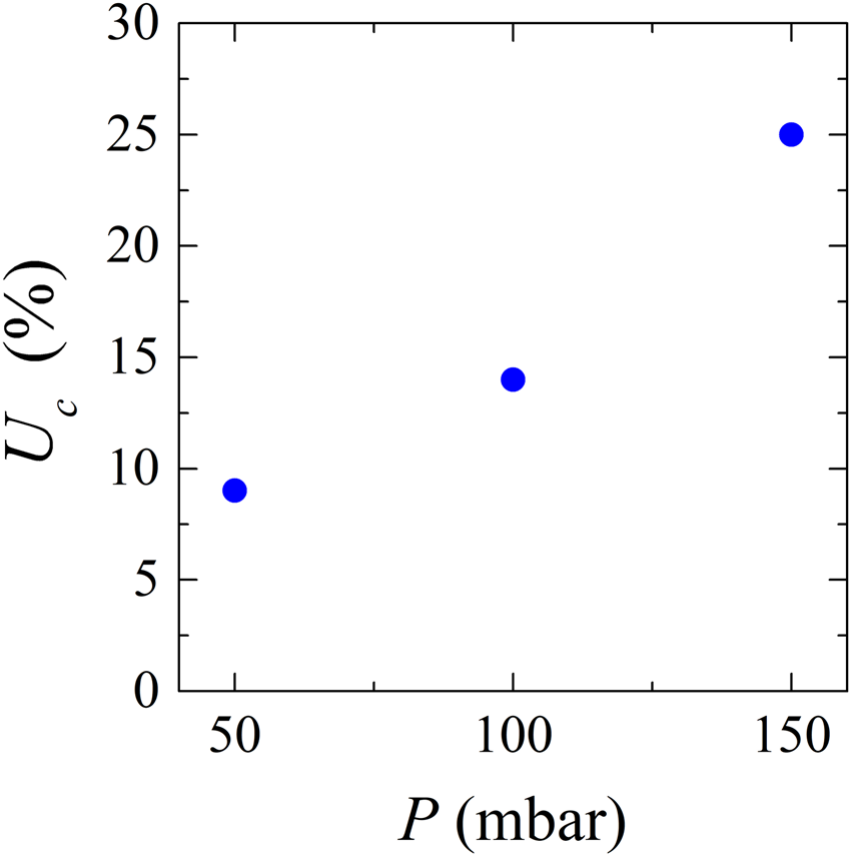
Percentage of unstable clogs (*U*_*c*_) for different system pressures (*P*).

As is clear from the previous section, it is not uncommon for microgels to be pushed all the way through the pores. Sometimes, a pore is blocked but after some time the clog is released, leaving the channel open until another microgel clogs the pore again. In the experiments carried out at different pressures we observed that most of the clogs are stable, meaning that a clog formed will remain in the channel and at the same position until the end of the experiment. The percentage of clogs that are pushed through is dependent on the applied system pressure (Fig. 5) and is as high as 25% at the highest applied pressure. The percentage is calculated by dividing the number of clogs that were released by the total number of observed clogs and these values are not dependent on the duration of the filtration run (5–10 minutes).

### Cake behavior

**Figure 6:**
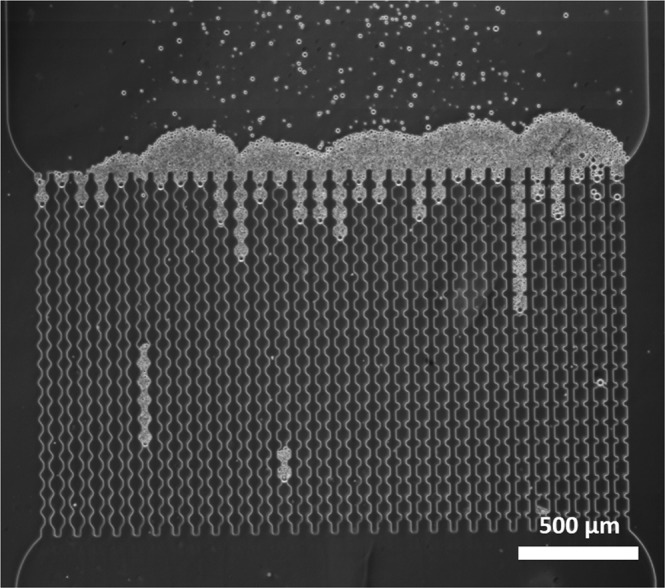
Cake layer forming on top of the channels as a result of channel clogging and subsequent particle deposition.

In the microfluidic membrane system, microgels do not block the constriction for liquid flow^29^, since microgel particles are still carried toward the channels after the system is clogged where they start to accumulate, forming a cake layer (Fig. 6). We keep the microgel suspension flowing until a cake of approximately 250 µm height is formed, and next investigate the extent of compression/deformation. The situation in Fig. 6 is depicted after the device has been clogged and more particles have accumulated over a period of time. Depending on the efficiency of primary clogging, a pore may not carry any liquid (so no additional cake) or still be open for liquid transfer which leads to considerable layer formation and great differences in thickness over the width of the device.

Cake compression and relaxation experiments were done by gradually changing the applied pressure and analyzing the resulting images. The changes in pressure are gradual and do not provoke clog instability. The cake reaches steady state very rapidly, in a matter of seconds after a change of pressure, and recompression is almost totally reversible, since the area of the cake is almost the same as before relaxation (see supporting information). The actual difference in size may be influenced by some particles arriving at the cake layer, but since the swelling/deswelling processes are fast it can be expected that this only played a minor role if any.

**Figure 7:**
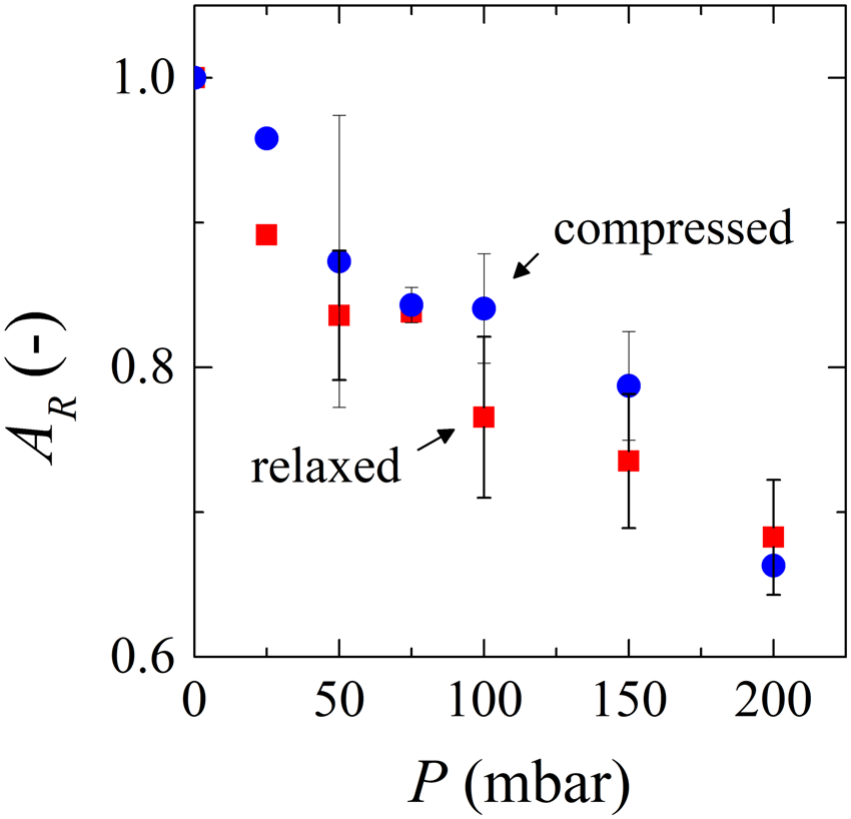
Relative cake area (*A*_*R*_) as a function of pressure (*P*) for relaxation (red squares) and compression (blue circles). Data for each pressure correspond to independent experiments.

To compare all relaxation/compression experiments we calculate the relative area by dividing the final cake area by the initial cake area. We replotted the relative area against the pressure applied for relaxation and compression (Fig. 7). Please note that the data for each pressure shown in the graph correspond to an independent experiment.

The relaxation curve seems a little lower than the compression curve (possibly as a result of particle-particle adhesion), but when we take the error bars into account we can consider that both curves overlap. As mentioned, the system compresses and decompresses in the same degree; we are looking at the thermodynamic state. When considering the values in the graph, it is clear that the volume reduction of particles at these pressures is considerable (>30% at 200 mbar; the area in the graphs can be interpreted as a volume given the depth of the chip). This corresponds to a diameter change of 10% for 10 micrometer particles, which is sufficient to influence filtration considerably even at pressures that are used in practice for microfiltration.

Total reversibility of cakes formed by compressible particles such as wastewater sludge flocs has also been described^30^, although some studies claim that total reversibility of compressible particles only means that the particles have not been compressed long/hard enough to form a cohesive cake layer^30–32^. We investigate this further using the microfluidic centrifugation methods.

### Centrifugation

**Figure 8:**
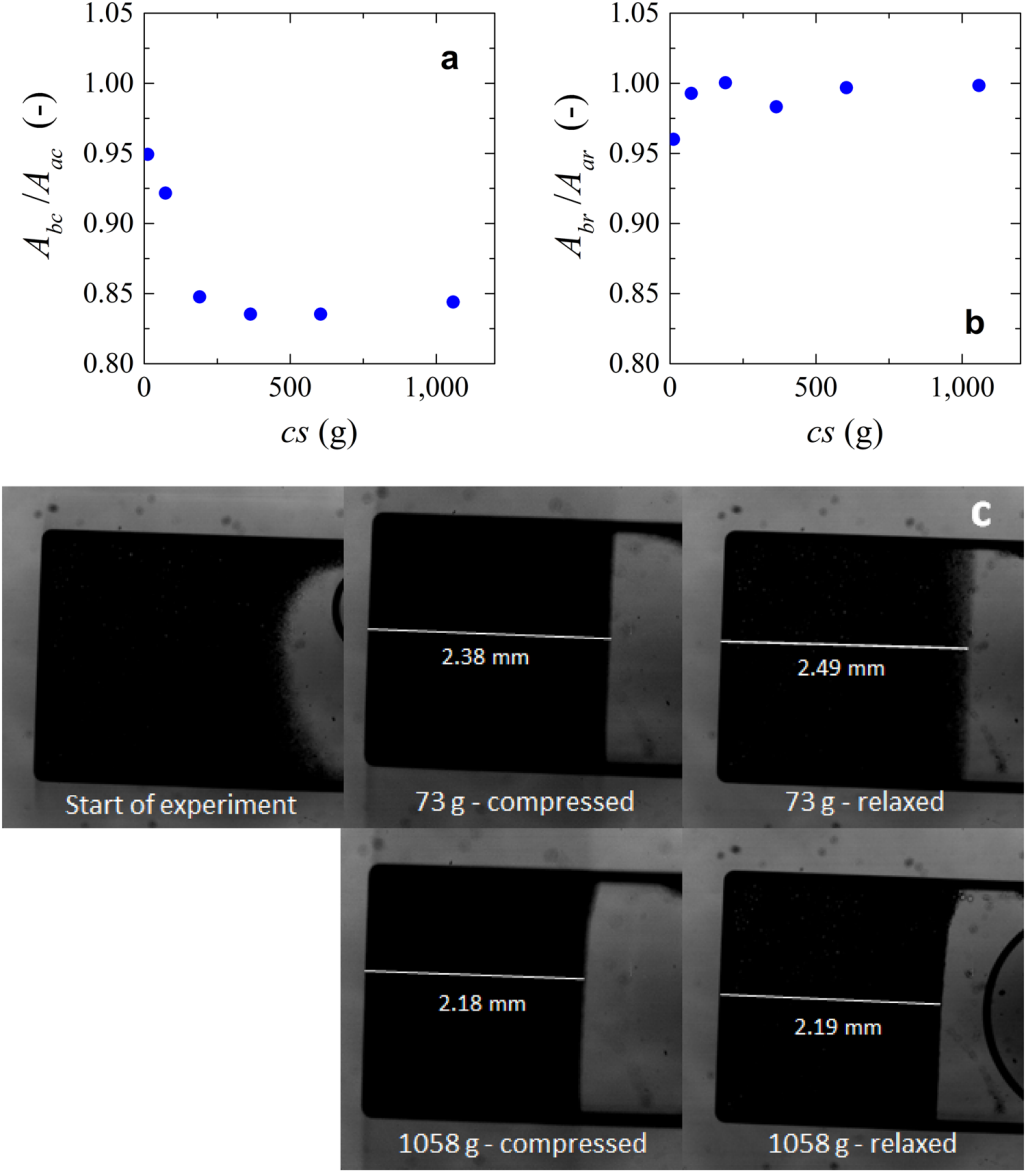
(**a**) Compression data of microgels. Ratio between area after compression (*A*_*ac*_) and the area before compression (*A*_*bc*_) versus centrifugation speed (*cs*). (**b**) Relaxation data of microgels. Ratio between area after (*A*_*ar*_) and before relaxation (*A*_*br*_) versus centrifugation speed (*cs*). (**c**) images showing the compressed and relaxed microgels at low and high number of g-forces. Values of area were measured manually with an average measurement error <0.5%.

We use centrifugation experiments to simulate the compression and relaxation of the microgels when subjected to compressive forces only. The results shown in Fig. 8 relate to the experiments done with the rectangular microchip chamber. The compression of the microgels at low g-force seems similar to compression of cake layers in the microfluidic devices, in which a steep decrease in area is followed by a constant cake/deposit area (supporting information).

During the relaxation experiments, we found that the extent to which the deposit relaxes depends on the applied g-force. A certain degree of relaxation can be observed in experiments in which the applied force was low (Fig. 8b, and top images in c), but as the microgels are exposed to more and more force, the reversibility of the deposit is almost nonexistent within the experimental time frame (Fig. 8c, bottom images). The maximum centrifugation speed where we can see some relaxation corresponds to a pressure of 36 mbar, whereas in the model microfiltration experiments, full relaxation was observed at pressures up to 200 mbar (Fig. 7). The plateau could be a combined result of compression behavior and changing the packing structure. However, in these experiments we did not have means to distinguish between both effects and have taken them as one. Since the same particles were used in both centrifugation and microfiltration experiments, the question is what causes the difference.

**Figure 9:**
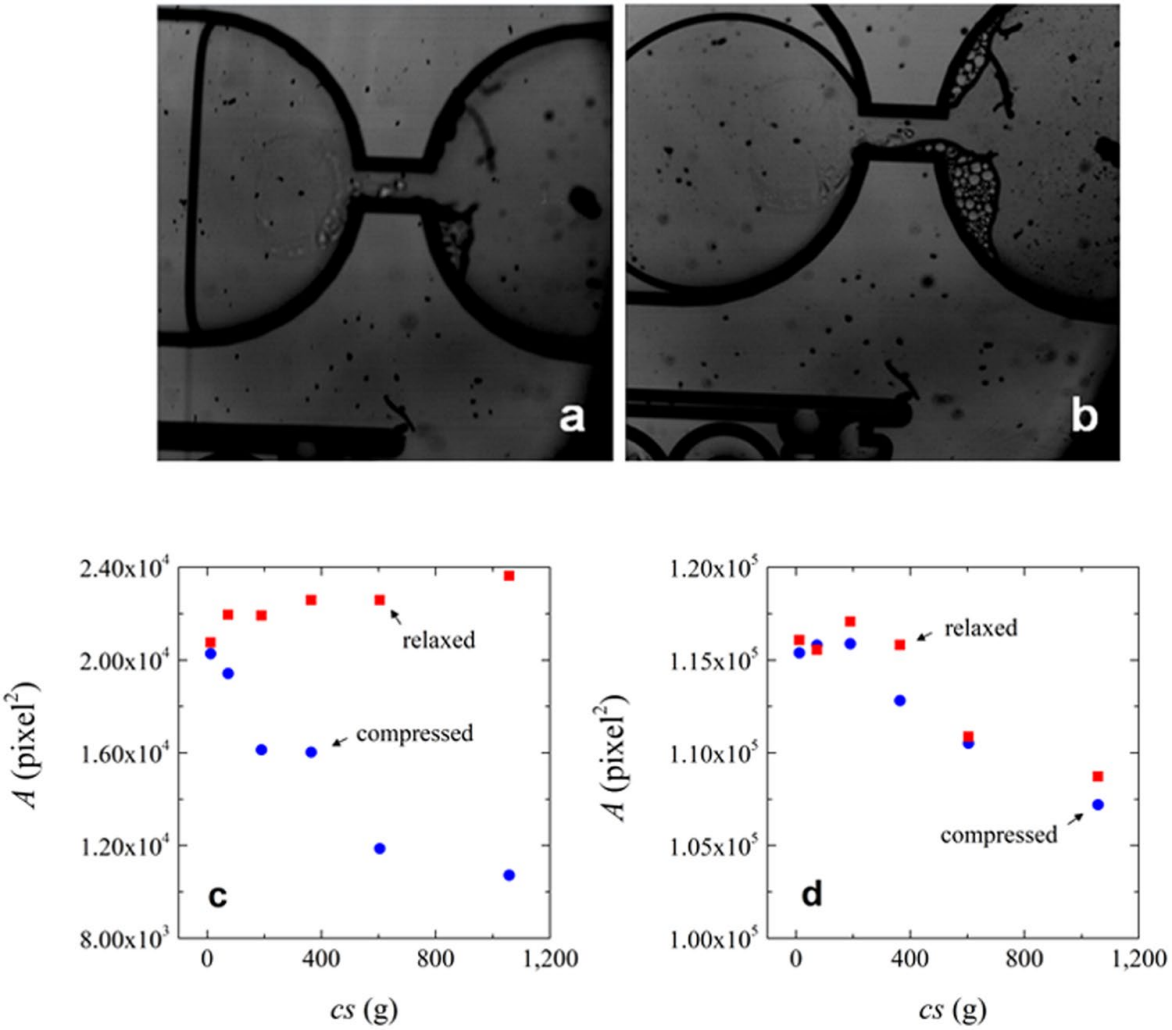
Area of Sephadex G100 microgels in the hourglass configuration microchips (**a**) At 1058 g. (**b**) After 5 minutes of relaxation. Area of the microgels as function of the centrifugal speed at (**c**) hourglass chamber configuration and (**d**) squared chamber configuration. Please note that each centrifugal speed corresponds to an individual experiment. Blue circles represent compressed microgels and orange squares correspond to relaxed microgels; please note that in all cases Sephadex was used. Values of area were measured manually with an average measurement error <0.5%.

To answer this question, we carried out centrifugation experiments with the hourglass configuration chamber that has two areas connected by a small channel, as if it was a single pore, but of course with bigger dimensions. We tried to use the same microgels as for the previous experiments but the microgels quickly transfer to the bottom of the chamber as soon as the g force is increased, leading to the same behavior as observed in the square chamber. That is why we opted for the use of the larger Sephadex microgels, of which a part stays in the first section of the chamber (so, before the “pore”) allowing us to observe “cake “ behavior; please note that on the pictures of Fig. 9, the right chamber contains the deposit and the left chamber is partly filled with water.

Figure 9a,b show that for the Sephadex particles in the hourglass configuration, total relaxation of the deposit occurs even at the highest force used (1058 g), whereas this is not the case in the rectangular chamber. The difference between the square and hourglass configurations is related to the freedom that the compressed material has to reswell. This can be seen as the availability of solvent for capillary effects and also the fact that there is no layer of water above the deposit in the hourglass configuration to be “pushed” away. For this reason, the deposit can reswell.

This also implies that full relaxation can be influenced by the design of the channel, a.k.a. the pore geometry. Furthermore, we expect these effects to be relevant for cake layers that build on top of membranes that, depending on the pore geometry can be removed more easily. As discussed before, for the microfluidic filtration system, even after the formation of the cake layer, there is still a flow of water between the microgels. This flow might avoid permanent/strong interactions between the particles as long as the applied pressure does not deform the particles too much. To be complete, differences in g-forces due to the size of the cell are not expected to have played a role in our experiments.

### Implications for membrane filtration

When considering our findings in the light of membrane filtration, it is clear that large soft particles can clog pores effectively, but also can be pushed through the pores depending on many factors such as particle size, polydispersity of the particle suspension, applied pressure and pore configuration. Although currently not taken as a starting point for membrane process design, cake formation and behavior can be reversible depending on the applied pressure, and to some extent layer thickness. We expect that cake reversibility is an important parameter that co-determines removal of a cake layer by backwashing, and through that as well membrane life time.

With the use of centrifugation systems we are able to extend our experiments to higher pressures, which could be of interest to for example ultrafiltration, nanofiltration, and reverse osmosis applications. It is also good to mention that the particle properties can be systematically varied, and the propensity of deswelling/deformation charted using the microfluidic systems presented here.

## Conclusions

In this paper, we investigated the behavior of soft microgels in microfluidic filtration systems as well as in centrifugation systems in order to emulate various situations occurring during membrane filtration. The propensity to pore clogging, and the position (depth) at which this takes place in a model membrane, depends on the applied pressure. At low pressure pores block immediately, while at higher pressures microgels deform and can be pushed through pores that are smaller than the microgel diameter, to potentially block pores deeper in the model membrane. At even higher pressure, particles can be more and more pushed through the pores, even after initially blocking them.

When forming a cake layer on top of the model membrane, we found that cake compression may be as high as 30% but it is totally reversible. These results are in line with those obtained with the so-called hourglass configuration in which a thin layer of microgels was present, but are in complete contrast with results obtained with a rectangular configuration containing a thick layer of microgels that compressed irreversibly. This shows that the presence of water near the microgels is of essence for reversibility of cake/deposits.

Our results are useful for the understanding of membrane filtration of compressible particles and shed a new light on cake layers and their ease of removal. We clearly showed that soft particles can be pushed through pores that are smaller than their original dimension, and deformation/deswelling should be taken into consideration for membrane design and process optimization. The extent to which this takes place can be quantified using the model systems that we presented here.

## Supplementary information

Cake compression and relaxation
