# Supplementary material for: Microfluidic model systems used to emulate processes occurring during soft particle filtration
Source: Sci Rep. 2019 Feb 28;9:3063. doi: 10.1038/s41598-019-39820-z (PMC6395687; doi:10.1038/s41598-019-39820-z)
Supplement: Supplementary file 1 — Cake compression and relaxation [file 41598_2019_39820_MOESM1_ESM.docx]

Microfluidic model systems used to emulate processes occurring during soft particle filtration.

Izabella Bouhid de Aguiar^1,2^, Martine Meireles^2^, Antoine Bouchoux^3^, Karin Schroën^1^

^1^ Laboratory of Food Process Engineering, Wageningen University, Wageningen, the Netherlands.

^2^ Laboratoire de Génie Chimique, Université de Toulouse, CNRS, INPT, UPS, France

^3^ Laboratoire d’Ingénierie des Systèmes Biologiques et des Procédés, CNRS, INRA, INSAT, Université de Toulouse, 31400, France

Supporting Information


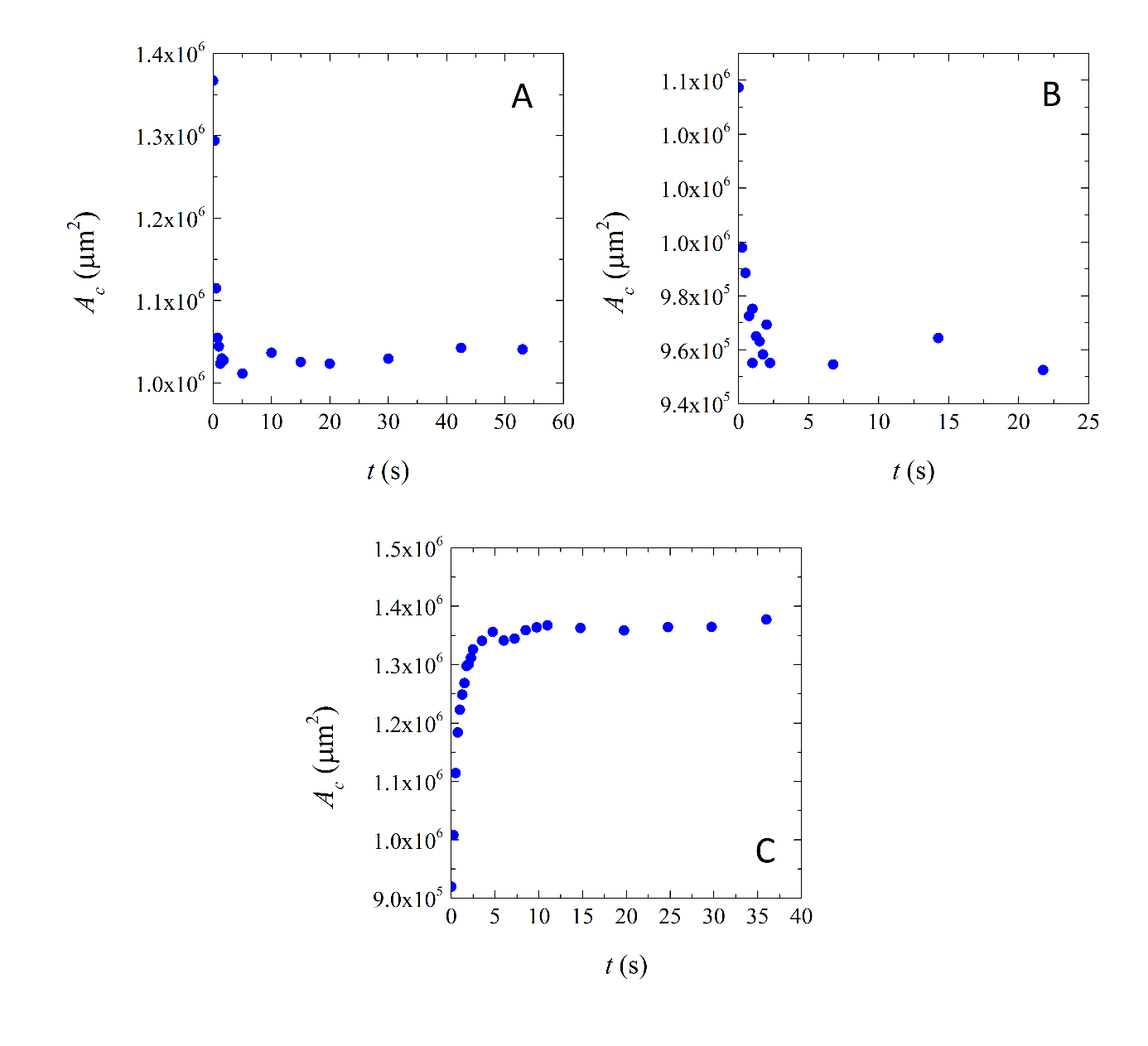


Figure S1. Cake area (Ac) as a function of time (t) A) from 0 to 50 mbar, B) from 50 to 100 mbar and C) from 100 to 0 mbar.
